# Transient global amnesia: 7 Tesla MRI reveals more hippocampal lesions with diffusion restriction compared to 1.5 and 3 Tesla MRI

**DOI:** 10.1007/s00234-022-02998-7

**Published:** 2022-06-27

**Authors:** Runa Geirmundsdatter Unsgård, Thanh P. Doan, Knut Kristian Nordlid, Kjell Arne Kvistad, Pål Erik Goa, Erik Magnus Berntsen

**Affiliations:** 1 Department of Circulation and Medical Imaging, Faculty of Medicine and Health Sciences, Norwegian University of Science and Technology, Trondheim, Norway; 2 Present Address: Department of Radiology and Nuclear Medicine, St. Olavs Hospital, Trondheim University Hospital, Trondheim, Norway; 3 Department of Neurology and Clinical Neurophysiology, St. Olavs Hospital, Trondheim University Hospital, Trondheim, Norway; 4 Department of Neuromedicine and Movement Science, Norwegian University of Science and Technology, Trondheim, Norway; 5 Department of Physics, Faculty of Natural Sciences, Norwegian University of Science and Technology, Trondheim, Norway

**Keywords:** 7 T, 7 T MRI, TGA, DWI lesion, Hippocampal lesion

## Abstract

**Purpose:**

To assess the ability of 7 T MRI to detect hippocampal DWI lesions in the acute phase of TGA compared to 1.5 T/3 T MRI.

**Methods:**

Patients with a clinical diagnosis consistent with TGA and a 1.5/3 T MRI underwent an additional 7 T MRI when the 7 T system was available for clinical use, thus serving as their own controls.

**Results:**

Thirteen TGA patients with a median age of 68.5 years (range 46–77 years) were included and imaged at 1.5/3 T (median 17 h after onset of symptoms, range 3–23 h) and 7 T (median 23 h after onset, range 15–46 h). The 7 T MRIs were performed a median of 15 h after the 1.5/3 T MRIs (range 1–28 h). At 1.5/3 T, six patients (46%) were found to have at least one hippocampal DWI-lesions supporting the TGA diagnosis, which increased to 11 patients (85%) when examined at 7 T (*p* = 0.03). At 1.5/3 T, nine hippocampal DWI lesions were detected, which increased to 19 at 7 T, giving an increased detection rate of 111% (*p* = 0.002). Both neuroradiologists found the hippocampal DWI lesions at 7 T to have higher conspicuity and be easier to categorize as true findings compared to 1.5/3 T.

**Conclusion:**

Seven-Tesla MRI showed both a statistically significant increase in the total number of detected hippocampal DWI lesions and the proportion of patients with at least one hippocampal DWI lesion supporting the TGA diagnosis compared to 1.5/3 T. Clinical use of 7 T will increase the number of patients having their TGA diagnosis supported by MRI, which can be especially useful in patients with negative 1.5/3 T MRI and low clinical certainty.

## Introduction

Transient global amnesia (TGA) is a condition where patients experience a sudden onset of anterograde and retrograde amnesia lasting up to 24 h without focal neurological symptoms. Associated symptoms such as headache, nausea, and dizziness can last longer than the amnestic period [1]. Patients fully recover from the condition without long-term complications, but the recurrence rate is 6–10% [2, 3]. The study with the longest follow-up included 51 TGA patients over 7 years, finding a recurrence rate of 8% [4]. There is a higher incidence of migraine with and without aura in patients with TGA compared to healthy controls [3, 5–7]. One study found migraine as a risk factor for TGA in patients younger than 56 years [6]. The underlying mechanism causing TGA is unelucidated, and the literature suggests etiologies such as migraine-related mechanisms, hypoxic-ischemic events, venous flow abnormalities, psychological stressors, and epilepsy-related activity [1]. The pathophysiology seems to involve a cascade of events affecting the neurons of the cornu ammonis 1 (CA1) region of the hippocampus, which are thought to be more vulnerable to various types of biochemical stressors [1, 8].

MRI is the imaging modality of choice to rule out other conditions and support the TGA diagnosis. In many TGA patients, MRI reveals unilateral or bilateral punctate areas of restricted diffusion in the CA1 region of the hippocampus on diffusion-weighted imaging (DWI), which also may be visible on T2-weighted images [7, 9, 10]. In rare cases, extra-hippocampal lesions with diffusion restriction have been described, often attributed to ischemia, but recent publication has raised some controversy about the pathogenesis of these lesions [11]. It has been shown that the hippocampal diffusion changes are detectable as early as 6 h after symptom onset, but the detectability increases and reaches a maximum after 12 to 24 h, lasting up to 96 h [7, 9, 10, 12–15]. A recent systematic review found a plateau phase for the detection rate between 12 and 96 h of approximately 70%, while a large single-center study indicates that the 12–24 h time window is the most ideal with a detection rate of 93%, using a tailor-made DWI sequence [14, 15]. The T2 changes succeed IN the diffusion changes, having a maximum detectability after 24 h [10]. Both the DWI and T2 findings are reversible within 10 days, with no detectable remnant or sequela even when imaged at 7 T [10, 16].

The detection rate of the hippocampal DWI-lesions varies from 0 to 100% between different studies due to both differing imaging parameters and timing of imaging after symptom onset [15]. Recent studies show a detection rate of 70% between 12 and 96 h, still leaving 30% of TGA patients without detected hippocampal DWI lesions [12, 14]. All previous studies with TGA patients imaged within 96 h have been performed on 1.5 T or 3 T MRI systems, and no TGA lesion imaged at 7 T has previously been published. 7 T has approximately 2.3 times higher signal-to-noise ratio (SNR) than 3 T, which can be used to give higher spatial resolution and, at the same time, better conspicuity of findings, allowing identification of more subtle changes [17–19]. Thus, imaging TGA patients at 7 T could potentially identify more hippocampal lesions with diffusion restriction compared to 1.5 T and 3 T. However, due to shorter T2-relaxation times and higher sensitivity to susceptibility-related artifacts at 7 T, the clinical value of DWI at 7 T compared to 1.5 T/3 T is not well established [20].

The aim of this study was to assess the ability of 7 T MRI to detect hippocampal DWI lesions in TGA patients compared to clinical 1.5/3 T MRI in individual patients serving as their own controls.

## Material and methods

This is an observational study of patients with a clinical diagnosis of TGA hospitalized at a single regional center between October 2020 and October 2021. Patients with a clinical diagnosis consistent with TGA and a 1.5/3 T MRI had an additional 7 T MRI during their stay when the 7 T system was available for clinical use, irrespective of whether any hippocampal DWI lesions had been detected at the standard clinical 1.5/3 T MRI or not. Thus, the study cohort was randomly composed based on the availability of the 7 T MRI system. The time of symptom onset was collected from the patients’ medical charts and recorded as the time from witnessed onset. In cases with an uncertain time of onset, the time of contact with primary healthcare was used. The clinical diagnosis was based on the seminal criteria of Hodges and Warlow, with additional attention given to the spectrum of differential diagnoses [21, 22]. Patients gave their informed consent to 7 T imaging and research, which was also approved by the regional ethics committee (reference 108066).

### MRI protocol

Imaging at 1.5/3 T was performed according to clinical routine on different Siemens MRI systems (Siemens Healthineers, Erlangen, Germany), resulting in slightly differing acquisition parameters. DWI was acquired using a single-shot spin-echo planar-imaging sequence (SS SE-EPI) with *b*-values of 0, 500, and 1000 s/mm^2^ on the MAGNETOM Avanto and Sola systems, and using a RESOLVE sequence with *b*-values of 0 and 1000 s/mm^2^ on the MAGNETOM Avanto Fit and 3 T systems. Transversal DWI with AC-PC alignment was acquired with a slice thickness of 5 mm, a slice gap of 20%, and a pixel size of 1.20 × 1.20 to 1.40 × 1.40 mm^2^ at 1.5 T (except in two cases where zero filling interpolation was used, giving pixel size 0.60 × 0.60 mm^2^) and a slice thickness of 3 mm, slice gap of 10–20% and pixel-size 1.33 × 1.33 to 1.38 × 1.38 mm^2^ at 3 T (none using zero filling interpolation). Most of the patients also had a dedicated DWI sequence aligned perpendicular to the hippocampal long axis with a slice thickness of 3 mm, slice spacing of 10%, and a pixel size of 0.72 × 0.72 to 0.78 × 0.78 mm^2^ at 1.5 T (all using zero filling interpolation except in two cases where pixel size was 1.44 × 1.44 mm^2^) and a slice thickness of 2 mm, slice gap of 10%, and a pixel-size of 1.15 × 1.15 to 1.38 × 1.38 mm^2^ at 3 T (none using zero filling interpolation). Furthermore, most patients had a coronal T2 aligned perpendicular to the hippocampal long axis with a slice thickness of 2.0–3.0 mm, slice-gap of 10%, and pixel size 0.24 × 0.24 to 0.51 × 0.51 mm^2^.

Imaging at 7 T was performed on a Siemens MAGNETOM Terra system in clinical mode using a 1Tx32Rx head coil (Nova Medical, Inc.), both CE labeled and FDA approved for clinical use. Transversal DWI was acquired using an AC-PC aligned RESOLVE sequence with *b*-values of 0 and 1000 s/mm^2^, slice thickness 2.00 mm, slice gap of 30% (one case 0%) and pixel size 0.50 × 0.50 mm^2^ [TR = 5020 ms (range 5000–5520 ms), TE = 46.2 ms (except one case TE 48.2 ms), FOV 230 × 230 mm, acquisition matrix 230 × 230 and reconstruction matrix 460 × 460], except in one case not using zero filling interpolation giving pixel size 1.02 × 1.02 mm^2^ [TR = 5120 ms, TE = 46.2 ms, FOV 229 × 229 mm, acquisition and reconstruction matrix 224 × 224]. Coronal DWI perpendicular to the hippocampal long axis was also acquired using a RESOLVE-sequence with *b*-values of 0 and 1000, slice thickness of 2.00 mm, slice gap of 10% (except one case 15% and one case 30%), and pixel size 1.02 × 1.02 mm^2^ [TR = 5700 ms, TE = 83.2 ms, FOV 229 × 229 mm, acquisition and reconstruction matrix 224 × 224]. Furthermore, coronal T2 images perpendicular to the hippocampal long axis with slice-thickness 1.00 mm, slice gap of 10%, and pixel-size of 0.25 × 0.25 mm^2^ were acquired using a turbo spin echo sequence [TR = 7950 ms (except in three cases), TE = 48 ms, 3 averages (except in two cases with 2 averages), FOV 220 × 220 mm, acquisition matrix 432 × 432 and reconstruction matrix 864 × 864].

### Image interpretation

For the purpose of this study, two neuroradiologists independently and blinded for each other’s interpretation investigated both the 1.5/3 T and 7 T examination for each patient once; the first neuroradiologist EDiNR board certified with 7 years of experience, and the second with more than 30 years of experience in neuroradiology. All DWI images were visually inspected with the PACS software used in our radiological department (Sectra IDS7, Sectra AB, and Sweden) to detect the presence and number of hippocampal lesions with restricted diffusion. All images were consistently interpreted using default settings in the PACS software with manual adjustment of window and level when needed.

### Statistics

A paired-samples t-test was used to detect any difference in the total number of hippocampal lesions between the 1.5/3 T and 7 T examinations. A McNemar’s mid-*p*-test was used to investigate whether the proportion of patients identified with hippocampal DWI lesions significantly changed after the 7 T examination, as this test has been recommended for paired binominal proportions [23, 24]. IBM SPSS statistics version 27 was used for statistical analysis.

## Results

Thirteen TGA patients (seven women) with a median age of 68.5 years (range 46–77 years) were included and imaged at both 1.5/3 T and 7 T. The 1.5/3 T examinations were performed with a median of 17 h after symptom onset (range 3–23 h), which for 11 patients was within the 12–24 h time window expected to have maximum detectability. In four patients, the time of symptom onset was unclear and set to the time of contact with healthcare. The 7 T examinations were performed with a median of 23 h after symptom onset (range 15–46 h) and with a median of 15 h after the 1.5/3 T examination (range 1–28 h). The two neuroradiologists came to the same conclusion in all patients when evaluating the presence and number of hippocampal DWI-lesions at both the 1.5/3 T and 7 T examinations for all patients. No extra-hippocampal DWI lesions were detected. These findings are summarized in Table 1.

**Table 1:** Descriptives for all patients, MRI examinations, and hippocampal DWI findings

Patient #/sex/age	Clinical MRI (Siemens)	Time between symptom onset and imaging (h)			Number of hippocampal DWI- lesions at clinical MRI		Number of hippocampal DWI- lesions at 7 Tesla MRI	
		1.5/3 T	7 T	Difference	Left	Right	Left	Right
1/M/68	MAGNETOM Avanto1.5 T	22	45	23	0	1	1	1
2/W/72	MAGNETOM Skyra3 T	23	25	2	1	1	1	1
3/W/72	MAGNETOM Sola1.5 T	17	22	5	0	0	0	0
4/W/46	MAGNETOM Prisma3 T	14	19	5	0	0	2	0
5/W/63	MAGNETOM Avanto1.5 T	15*	16*	1	0	0	0	0
6/W/71	MAGNETOM Sola1.5 T	16	40	23	1	0	1	1
7/M/77	Biograph mMR3 T	18	19	1	1	1	1	1
8/M/71	MAGNETOM Prisma3 T	7*	22*	15	0	0	1	0
9/M/66	MAGNETOM Avanto Fit1.5 T	3*	23*	20	0	0	1	0
10/M/66	Biograph mMR3 T	20	43	23	0	0	2	0
11/W/69	MAGNETOM Avanto Fit1.5 T	20*	43*	23	1	0	1	0
12/M/68	Biograph mMR3 T	14	15	1	2	0	2	1
13/W/74	MAGNETOM Avanto1.5 T	18	46	28	0	0	0	1
	Number of DWI lesions				6	3	13	6
	Total				9		19	
	Number of patients				5	3	10	6
	Total				6		11	

^*#*^: patient number; *M: man*; *W: woman*, ; *Age*: in years; *: time from first contact with healthcare, no exact time of symptom onset is known

### Detection rate

At 1.5/3 T, six of the 13 patients were found to have at least one hippocampal DWI lesions supporting the TGA diagnosis, giving a detection rate of 46% for all 13 patients and 55% for the eleven patients within the 12–24 h time window. When imaged at 7 T, hippocampal DWI lesions were found in 11 patients, increasing the detection rate at 7 T to 85%. This constituted a significant difference in the proportion of patients with identified with hippocampal DWI lesions after the 7 T examination (*p* = 0.03, McNear’s mid-*p*-test).

**Fig. 1:**
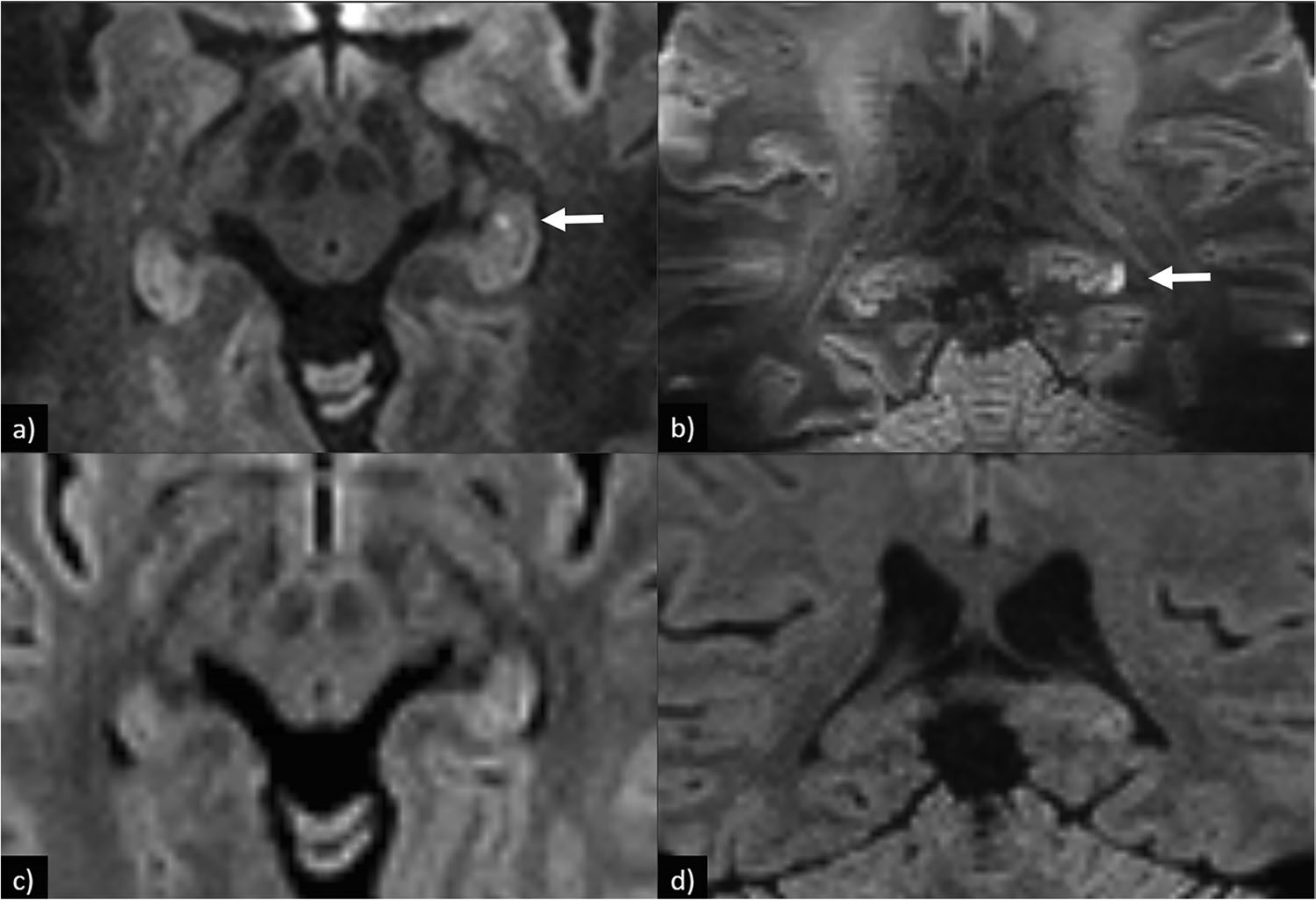
DWI for patient no. 4 with two left-sided hippocampal DWI-lesions at 7 T but not 3 T. All DWI are RESOLVE. **a** 7 T, DWI, transversal 2 mm, slice gap 0.6 mm, pixel size 0.50 × 0.50 mm^2^; **b** 7 T, DWI, coronal 2 mm, slice gap 0.2 mm, pixel size 1.02 × 1.02 mm^2^; **c** 3 T, DWI, transversal 3 mm, slice gap 0.6 mm, pixel size 1.33 × 1.33 mm^2^; **d** 3 T, DWI, coronal 2 mm, slice gap 0.2 mm, pixel size 1.15 × 1.15 mm^2^

**Fig. 2:**
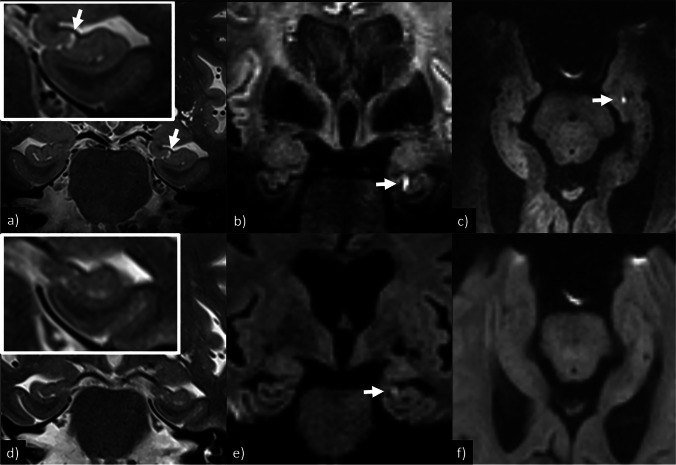
T2-weighted images and DWI for patient no. 11 show a left-sided hippocampal DWI-lesion barely visible on coronal 1.5 T DWI, but with clearly better conspicuity on coronal 7 T DWI which is also detectable on transversal 7 T DWI and coronal 7 T T2. All DWI sequences are RESOLVE. **a** 7 T, T2, coronal 1 mm, slice gap 0.1 mm, pixel size 0.25 × 0.25 mm^2^; **b** 7 T, DWI, coronal 2 mm, slice gap 0.2 mm, pixel size 1.02 × 1.02 mm^2^; **c** 7 T, DWI, transversal 2 mm, slice gap 0.6 mm, pixel size 0.50 × 0.50 mm^2^; **d** 1.5 T, T2, coronal 3 mm, slice gap 0.3 mm, pixel size 0.30 × 0.30 mm^2^; **e** 1.5 T, DWI, coronal 3 mm, slice gap 0.3 mm, pixel size 1.44 × 1.44 mm^2^; **f** 1.5 T, DWI, transversal 5 mm, slice gap 1.0 mm, pixel size 1.44 × 1.44 mm^2^

**Fig. 3:**
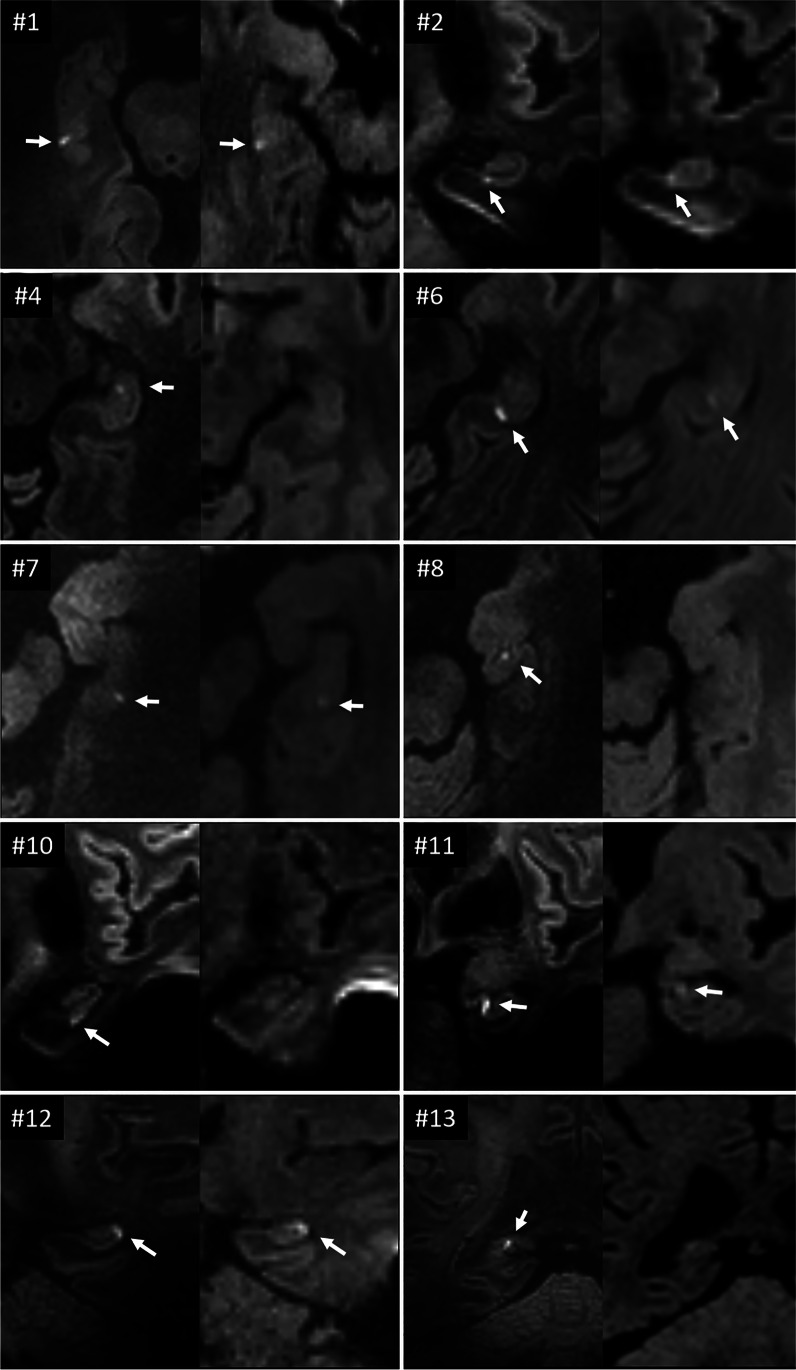
Corresponding DWI-images for 10 of the TGA patients, with the 7 T images to the left and the 1.5/3 T images to the right for each patient. The #-number corresponds to the patient number in Table 1. For patients 4, 8, 10, and 13, the DWI lesion was only detectable at 7 T. For the rest of the patients, the DWI lesions had higher conspicuity at 7 T

At 1.5/3 T, a total of nine hippocampal DWI lesions were detected, which increased to 19 lesions at 7 T, representing an increase of 111%. This constituted a significant increase in total lesions detected at 7 T (*p* = 0.002, paired-samples *t*-test). This significant increase in lesions at 7 T was present for both the 1.5 T and 3 T subgroup (*p* = 0.046 and *p* = 0.041, respectively). These findings are exemplified in Figs. 1, 2, and 3.

### Lateralisation

At 1.5/3 T, 50% of the patients with hippocampal DWI lesions lateralized to the left (3/6 patients), while 17% to the right (1/6 patients), and 33% bilaterally (2/6 patients). At 7 T this changed to 46% to the left (5/11 patients), 9% to the right (1/11 patients), and 45% bilaterally (5/11 patients). At 1.5/3 T, 67% of the hippocampal DWI lesions were found in the left hippocampus (6 vs 3 lesions), which changed to 68% (13 vs 6 lesions) at 7 T.

### Image interpretation

Both neuroradiologists independently judged the hippocampal DWI lesions easier to categorize as true findings due to higher conspicuity at 7 T, as shown in Figs. 2 and 3. It was also noted that it varied whether a hippocampal DWI lesion was best visible on the coronal or transversal DWI images. Likewise, hippocampal lesions were also more easily detectable on coronal T2 images at 7 T compared to 1.5/3 T, as shown in Fig. 2.

## Discussion

We have investigated the ability of DWI at 7 T to detect hippocampal lesions compared to DWI at 1.5/3 T in 13 patients in the acute phase of TGA. Each patient was imaged at both 1.5/3 T and 7 T, allowing us to use each of them as our own controls. We found that 7 T MRI significantly increased the number of patients with at least one hippocampal DWI-lesion supporting the clinical suspicion of TGA. Furthermore, the use of 7 T significantly increased the total number of detected hippocampal DWI lesions, revealing more patients with bilateral lesions than in previous studies.

### Detection rate

In our study, we found a detection rate for hippocampal DWI lesions supporting the TGA diagnosis of 46% (6/13 patients) for all patients and 55% (6/11 patients) for patients within the 12–24 h time window at 1.5/3 T. Four previous studies have reported data within this time window, of which our finding is comparable to Higashida et al.’s finding a detection rate of 40% (10/26 MRI scans) at 1.5/3 T [12]. Our result is, however, somewhat lower than in the study of Ryoo et al. with a detection rate of 67% (8/12 patients) at 1.5/3 T, the studies of Toledo et al. with a detection rate of 89% (8/9 patients) at 1.5 T and the study of Szabo et al. with a detection rate of 93% (approximately 25 MRI scans, inferred from Fig. 2) at 1.5/3 T [13, 14, 25]. These four studies were included in a systematic review giving a pooled sensitivity of 73% with a 95% confidence interval from 41 to 96%, bringing our results in line with previous literature. The discrepancy between these studies could partly be due to varying probability of the subjects having TGA, but is also likely caused by differing field strengths and imaging parameters, as increasing field strength and *b*-values, as well as decreasing slice-thickness, have shown to increase the detection rate [13, 22, 26]. Higashida et al. used either a standard SS SE-EPI sequence with a slice thickness of 5–8 mm and *b*-value of 1000 s/mm^2^ or an optimized SS SE-EPI sequence with a slice thickness of 2–3 mm and *b*-values of 1000 or 2000s/mm^2^ at 1.5 T and 3 T, finding that the optimized protocol more often used at 3 T gave an increased detection rate [12]. Ryoo et al. used a SS SE EPI with a slice thickness of 2 mm and a *b*-value of 2000s/mm^2^ at either 1.5 T or 3 T, finding a higher detection rate at 3 T (80% vs 57%) [13]. Toledo et al. used a DWI sequence not otherwise specified at 1.5 T [25]. Szabo et al. used a tailor-made DWI protocol (exact sequence not specified) parallel to the long axis of the hippocampus, with slice-thickness of 2 mm and *b*-values of 0, 1000, and 2000s/mm^2^ at either 1.5 T or 3 T [14]. However, none of these studies used the patients as their own controls, thus not showing these effects within the same patient, as we have.

In our study, when examining the same individuals at 7 T between 1 and 28 h after the 1.5/3 T examination, the detection rate of at least one hippocampal DWI lesion supporting the TGA diagnosis significantly increased to 85% (11/13 patients) (*p* = 0.03). Furthermore, the total number of hippocampal lesions increased from nine to nineteen, constituting a significant increase for the group as a whole (*p* = 0.002), as well as for both the 1.5 T and 3 T subgroup (*p* = 0.046 and *p* = 0.041, respectively). A possible confounder could be that the 7 T examinations were performed after the 1.5/3 T, giving time for additional lesions to manifest. This could be the case for the two patients initially imaged at 3 and 7 h. However, these two patients had an unknown time of onset of symptoms. Patients suffering from TGA usually present 6–8 h after symptom onset at the emergency ward according to our local registry. Consequently, it is possible that these two patients had already reached the 12–24 h window. The remaining 11 patients already had reached the optimal 12–24 h time window and plateau phase of detectability when imaged at 1.5/3 T. Thus, it is reasonable to attribute both the increase in the total number of hippocampal DWI lesions and the proportion of patients with detected hippocampal DWI lesions to the increase in magnetic field strength. This is further substantiated by the fact that several of the patients with additional hippocampal DWI lesions on 7 T had a very short time interval between their examinations, e.g., patient no. 4 with 5 h and patient no. 7 with 1 h. Both of these patients were initially examined at 3 T, with transversal and coronal RESOLVE-sequences with 2–3 mm slice thickness, making them highly comparable to the 7 T examination. The findings from patient no 4 are shown in Fig. 1. At last, we would like to call attention to the advantage that each patient served as their own controls, as it strengthens our findings.

While increased magnetic field strength generally provides higher SNR and thereby better image quality, this is not necessarily true for DWI [20]. One reason for this is the shortening of T2 at higher field strengths. In our study, this effect was partly compensated by the use of shorter TE at 7 T as compared to 1.5 T/3 T, which was enabled by a strong gradient system at 7 T. Furthermore, we chose to increase the spatial resolution somewhat compared to 1.5 T/3 T, but at the same time, take advantage of the increased SNR giving better conspicuity to the hippocampal DWI lesions. We also chose to include a coronal DWI sequence in both the 1.5/3 T and 7 T examinations to increase the detection rate. This is in line with the studies of Higashida et al., Ryoo et al., Szabo et al., and Kim et al., finding an increased detection rate when using a dedicated TGA protocol with increased image resolution, *b*-value, and field strength [12, 14, 27].

### Lateralisation

In our study at 7 T, 46% of the patients (5/11 patients) with hippocampal DWI lesions lateralized to the left, while 9% lateralized to the right (1/11 patients) and 45% bilaterally (5/11 patients). This is in line with the three largest studies to date, reporting lateralization to the left in 38–47% of patients with hippocampal DWI lesions. Szabo et al. had 272 patients of which, 41% lateralized to the left, 29% to the right, and 30% bilaterally [14]. Kim et al. had 96 patients of which 38% lateralized to the left, 29% to the right, and 33% bilaterally [27]. Higashida et al. had 79 patients, of which 47% lateralized to the left, 32% to the right, and 22% bilaterally [12]. Compared to these studies, we found fewer patients that lateralized only to the right, and more patients with bilateral lesions, this being a shift which we observed when moving from 1.5/3 T to 7 T. This could be due to the increased detection rate at 7 T, thus classifying more of the right lateralizing patients as bilateral instead, which at least was the case for one of our patients. Nevertheless, 68% of all hippocampal DWI lesions at 7 T in our study were found on the left side (13 vs 6 lesions). This left lateralization trend warrants future research into the pathophysiology behind TGA.

### Image interpretation

In addition to the higher detection rate of hippocampal DWI lesions at 7 T, we found that the lesions were easier to detect and categorize as true findings on 7 T images due to higher conspicuity, as shown in Figs. 2 and 3. Likewise, corresponding hippocampal lesions on coronal T2 were also easier to detect on the 7 T images. Both of these findings are likely due to the higher MRI-signal and image resolution following the increased SNR at 7 T. One confounding factor could be that the increased visibility at 7 T was an effect of the T2 lesions first appearing after the 1.5/3 T examination, as previous studies have reported them to be detectable first after 24 h [10]. However, in our 3 patients scanned at 7 T in the 12–24 h after onset of symptoms, it is possible to identify corresponding T2 lesions. Thus, the detectability of these T2 lesions also seems facilitated by increasing the magnetic field strength and relevant imaging parameters.

We also noticed that it varied whether a hippocampal lesion was best seen on the transversal or coronal DWI image at all field strengths, which could be due to the complex hippocampal CA1 anatomy. At 7 T, this could also be due to the slice-gap of 30% for the transversal DWI and 10% for the coronal DWI (except in two cases), which ranged from 0.2 to 0.6 mm. Since TGA lesions can be as small as 1–2 mm, there is a risk that such lesions could have disappeared within the gaps, or at least been blurred beyond detection by partial volume effects. These finding advocates for a refined tailor-made TGA protocol at 7 T without slice gap, keeping in mind the potential risk of introducing noise in adjacent slices.

### Clinical usefulness

Our findings show that 7 T MRI provides more support in establishing a TGA diagnosis than 1.5/3 T MRI and that 7 T MRI is useful in the evaluation of TGA patients in the acute and subacute setting, especially in patients with negative MRI at 1.5/3 T. Even though TGA is a clinical diagnosis, we believe the supportive findings of hippocampal DWI lesions in these patients are of direct clinical value. Firstly, there are other amnesic syndromes such as transient epileptic amnesia, psychogenic amnesic states, focal hippocampal stroke, diencephalic amnesia, and early stages of limbic encephalitis, which can be difficult to separate from TGA [24]. Secondly, it has previously also been shown by Szabo et al. that MRI can be particularly helpful when the clinical presentation is ambiguous, as they found that hippocampal DWI lesions supported the TGA diagnosis in 69% of patients with low clinical certainty [14]. Hence, 7 T MRI can provide support in even more uncertain cases than 1.5/3 T today. Thirdly, patients with TGA tend to have anxiety traits, which can be exacerbated by the lack of objective clinical and radiological findings [3, 28, 29]. Since TGA is considered self-limiting with a low recurrence rate, typical MRI findings are reinsuring in this patient population. Finally, imaging of these changes in the hippocampal areas is important to further understand the pathophysiology and eventually, hopefully, the cause of TGA [1].

### Strengths and limitations

One limitation in our study is the difference in magnetic field strength and varying scanning parameters in the standard clinical MRI examinations, as they have been done as part of the clinical routine. However, statistical analysis showed that there was a significant increase in the number of hippocampal DWI-lesions for both the 1.5 T and 3 T subgroups.

A second limitation in our study is the unknown time of symptom onset in four patients, as previous studies have found the hippocampal DWI lesions to have a maximum detectability plateau between 12 and 96 h [9, 10, 12–15]. As discussed, we do not believe that this affects our main findings since two of them had reached this plateau at their 1.5/3 T examinations, and a third one was assumed to have reached the plateau based on normal patient delay in this setting.

A third limitation is that the time interval between the 1.5/3 T and 7 T examinations varied from 1 to 28 h, thus possibly allowing more hippocampal DWI-lesions to appear. As discussed, we do not believe this significantly affects our main findings of more DWI lesions at 7 T, as exemplified with two of our patients where additional DWI lesions were discovered with an interval between examinations limited to 1 and 5 h. Nevertheless, it would be advantageous to reduce this time interval to a minimum in prospective studies, or even perform imaging at 3 T and 7 T in a mixed order to exclude this potential confounding factor.

A fourth limitation is that we have a relatively small number of patients being included based on the availability of the 7 T MRI system, thus it is uncertain how representative they are compared to a typical TGA population. However, it should be noted that our study has the major advantage that patients serve as their own controls, which precludes any inter-individual variability.

### Future possibilities

Future studies at 7 T should aim to reproduce our results in a larger cohort and minimize the time between examinations at different field strengths, preferably with 7 T scanning both before and after 3 T scanning in order to exclude timing as a cofound. Furthermore, protocols at both field strengths should be homogeneous and comparable. Additional advanced MRI techniques such as spectroscopy may be applied to elucidate the pathophysiological basis for TGA. Although 7 T MRI systems can almost exclusively be found at academic centers and university hospitals, we provide here evidence that they can be used clinically to support a diagnosis of TGA.

## Conclusion

We examined the same patients suffering from TGA with standard clinical MRI at 1.5/3 T and ultra-high-field MRI at 7 T. The use of 7 T MRI significantly increased the number of patients with at least one hippocampal DWI lesion supporting the TGA diagnosis by 83% (6 vs 11 patients) (*p* = 0.03). Imaging at 7 T also significantly increased the total number of detected hippocampal DWI lesions by 111% (9 vs 19 lesions) (*p* = 0.002). Clinical use of 7 T will increase the number of patients having their TGA diagnosis supported by MRI, which can be particularly useful in patients with negative DWI at 1.5/3 T and low clinical certainty.

## Data Availability

The authors confirm that all the data supports our published claims and comply with field standards.

## Code availability

The authors confirm that all MRI-examinations were reviewed on a standard clinical PACS system using Sectra Workstation (Sectra AB, Linköping, Sweden) and no other software or code was used.
